# Dataset of leachate volumes and surface areas for municipal solid waste (MSW) landfills in Ohio, USA from 1988–2020

**DOI:** 10.1016/j.dib.2023.108961

**Published:** 2023-02-09

**Authors:** Max J. Krause, Natalie Detwiler, William Eades, Davin Marro, Amy Schwarber, Thabet Tolaymat

**Affiliations:** a US Environmental Protection Agency Office of Research & Development, 26 Martin Luther King Dr W, Cincinnati, OH 45268 USA; b Oak Ridge Associated Universities, 26 Martin Luther King Dr W, Cincinnati, OH 45268 USA

**Keywords:** Municipal solid waste, Landfill, Leachate, Recirculation, Disposal

## Abstract

This data brief presents leachate disposal and management data for 43 active or closed municipal solid waste (MSW) landfills and planar surface areas for 40 of those landfills in Ohio, USA. Data were extracted from publicly available Annual Operational Reports from the Ohio Environmental Protection Agency (Ohio EPA) and consolidated into a digital dataset of two delimited text files. A total of 9,985 data points represent monthly leachate disposal totals, arranged by management type and by landfill. Leachate management data for some landfills extend from 1988-2020 but are mostly limited to 2010-2020. Annual planar surface areas were identified from topographic maps in the annual reports. A total of 610 data points were created for the annual surface area dataset. This dataset aggregates and organizes the information, allowing for accessibility and increased application to engineering analysis and research projects.

**Specifications Table**

**Table:** 

Subject	Environmental Science
Specific subject area	Waste Management
Type of data	Delimited text file (.csv)
How the data were acquired	Municipal Solid Waste (MSW) landfill owners submit annual reports to Ohio Environmental Protection Agency (Ohio EPA) which contain details about the facility's operations including amounts of waste collected, changes in facility capacity, volumes of leachate generated, and current maps of the landfill. The reports are housed in a public records repository (Ohio EPA eDocument Search, http://edocpub.epa.ohio.gov/publicportal/edochome.aspx). Reports were downloaded individually by facility identification number. Monthly landfill leachate volumes and management descriptions for 43 active or closed landfills were manually digitized by transcribing each data point into a delimited text file. Annual landfill surface areas were derived from topographic maps within the reports. Surface areas were determined using measurement tools in Adobe Acrobat Pro and Google Earth Pro. The data were examined by a separate individual to ensure transcription/measurement accuracy.
Data format	Raw
Description of data collection	Data were collected from MSW landfills (i.e., non-hazardous waste landfills) permitted in Ohio, USA. Construction and demolition debris or hazardous waste landfills were not included. The data collection includes monthly volumes of generated, disposed, and/or recycled MSW landfill leachate (i.e., wastewater generated from landfills) and annual surface areas from up to 43 landfills from 1988-2020. The data include leachate management descriptions and locations of disposal, if available.
Data source location	Primary data source: Ohio EPA eDocument Search (http://edocpub.epa.ohio.gov/publicportal/edochome.aspx)
Data accessibility	Repository name: Mendeley Data Direct URL to data: https://data.mendeley.com/datasets/rmn843c3bk/4 DOI: 10.17632/rmn843c3bk.4

## Value of the Data

• This dataset consolidates and digitizes publicly available leachate disposal and management data from landfills in Ohio, USA, making this information more accessible and easier to apply to research projects.
• The data can be useful for landfill operators, regulators, and engineers and scientists in the waste management field.
• The dataset includes surface areas for each landfill for normalization of leachate production rates.
• The data are useful for understanding long-term leachate generation trends, estimating internal landfill moisture content, calculating landfill operational costs, or making comparisons to other waste management technologies using life cycle assessments.

## 1 Objective

Landfill owners/operators in Ohio, USA must submit annual reports to the state regulatory agency, Ohio Environmental Protection Agency (Ohio EPA). Among other things, these reports include monthly volumes of leachate generated, disposed, or recycled and topographic maps that delineate the areas which contain waste. These reports are in electronic format (.pdf) but the data are not readily usable. This effort digitized and consolidated the monthly leachate volumes and annual surface areas into two separate delimited text files [1]. In solid waste engineering, when comparisons across landfills of different sizes are necessary, surface areas are used to normalize leachate generation rates (e.g., gallons per acre-day). The publication of this dataset increases accessibility and opportunities for studies regarding leachate generation, disposal, and/or recirculation. The dataset is integral to research that assesses the relative contributions to overall landfill moisture content, which has further implications for structural integrity, landfill gas collection, and facility costs.

## 2 Data Description

**Fig. 1:**
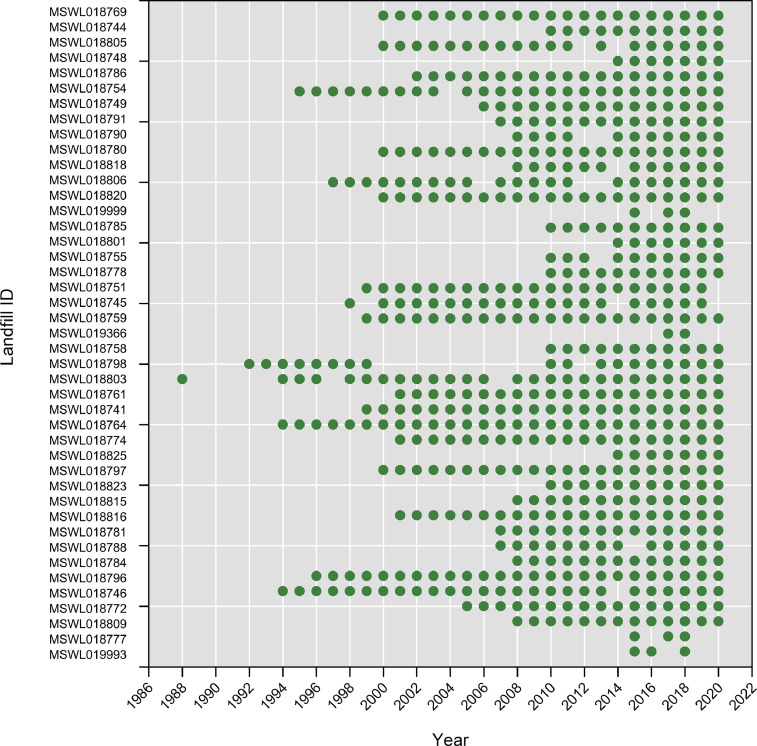
Green circle symbols indicate the presence of available leachate data in each year for each landfill, identified by its facility identification number.

The leachate data are categorized in a delimited text file format (Ohio_Leachate_Volumes.csv), organized under header columns: (A) Year, (B) Month, (C) Landfill Name, (D) Identification Number (i.e., Landfill ID), (E) Date, (F) Leachate Volume (gallons), (G) Management Type, (H) Management Description, (I) Location, and (J) Notes. Fig. 1 presents the distribution of leachate data within Ohio_Leachate_Volumes.csv indicating the presence or absence of data for each landfill by year. The availability of reports increased in more recent years.

Table 1 defines all Management Types and Management Descriptions used in the leachate dataset. Leachate is generated from landfills by the percolation of water through the waste layers over time. Water enters the landfill from the different types of waste, such as MSW, that the facility accepts, and from the infiltration of rain and snowmelt into the waste mass. Leachate is generally collected in a central holding tank or pond and then hauled via truck or discharged via sewer line to a wastewater treatment facility. The constraints on leachate disposal can be both economic and regulatory, thus, not all leachate that is generated is necessarily disposed in the same month. Alternately, leachate can be recycled back into the landfill via several different methods. This delays disposal costs and enhances microbial activity, waste decay, and gas generation which can also be economically beneficial [2]. Finally, leachate may be treated on-site or stored in a holding tank or pond. Ohio landfills are required to report the volume of leachate “managed” which historically meant disposed and/or recycled. Thus, most entries reflect volumes of leachate managed in these ways.

**Table 1:** Terminology definitions for leachate management types and descriptions.

Management Type	Definition	Management Description	Definition
Generated	Leachate that is generated on-site. The amount generated may or may not be greater than disposed quantities because leachate may be stored and disposed at different times.	Not applicable	Not applicable
Disposed	Leachate this is disposed offsite, usually to a municipal wastewater treatment plant	Discharged	Leachate that is discharged off-site via sewer line, force main, etc.
		Hauled	Leachate that is hauled off-site via tanker truck.
Recycled	Leachate that is re-introduced into the landfill, either through recirculation, solidification, or other means.	Recirculated	Leachate that is pumped into the landfill subsurface.
		Solidified	Leachate that is mixed with ash, wood chips, or other porous wastes to "solidify" the liquid before adding back into the landfill.
		Sprayback	Leachate that is sprayed onto the working face or other areas of the landfill surface.
Treated On-Site	Leachate that is treated on-site. This may be pre-treatment or complete treatment	Evaporated	Leachate that is evaporated on-site either through an active or passive system.
Stored	Leachate that is stored on-site in ponds, sumps, or tanks.	Not applicable	Not applicable

**Fig. 2:**
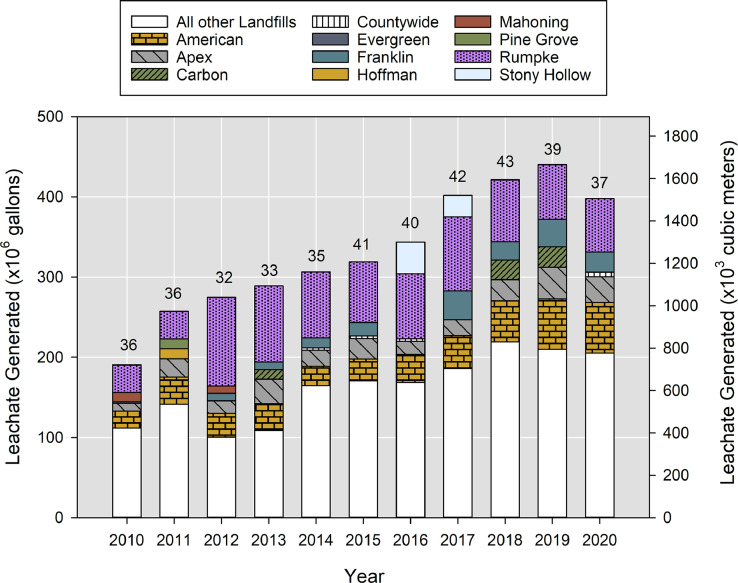
Reported leachate generation from 43 Ohio landfills in years 2010-2020. The number above the column indicates the number of sites for which data was found for that year.

Using data from Ohio_Leachate_Volumes.csv, Fig. 2 presents the total reported volume of leachate generated (taken as volume disposed, recycled, stored, or treated) from Ohio landfills, from 2010-2020. In the most recent years, Ohio landfills generated over 400 million gallons (1.6 million m^3^) of leachate each year. In these years, the top 5 leachate-generating sites accounted for 41-62% of the total leachate volume in Ohio. Rumpke Sanitary Landfill, the largest landfill by tons of waste disposed, contributed 13-40% of the total leachate volume in those 11 years.

**Fig. 3:**
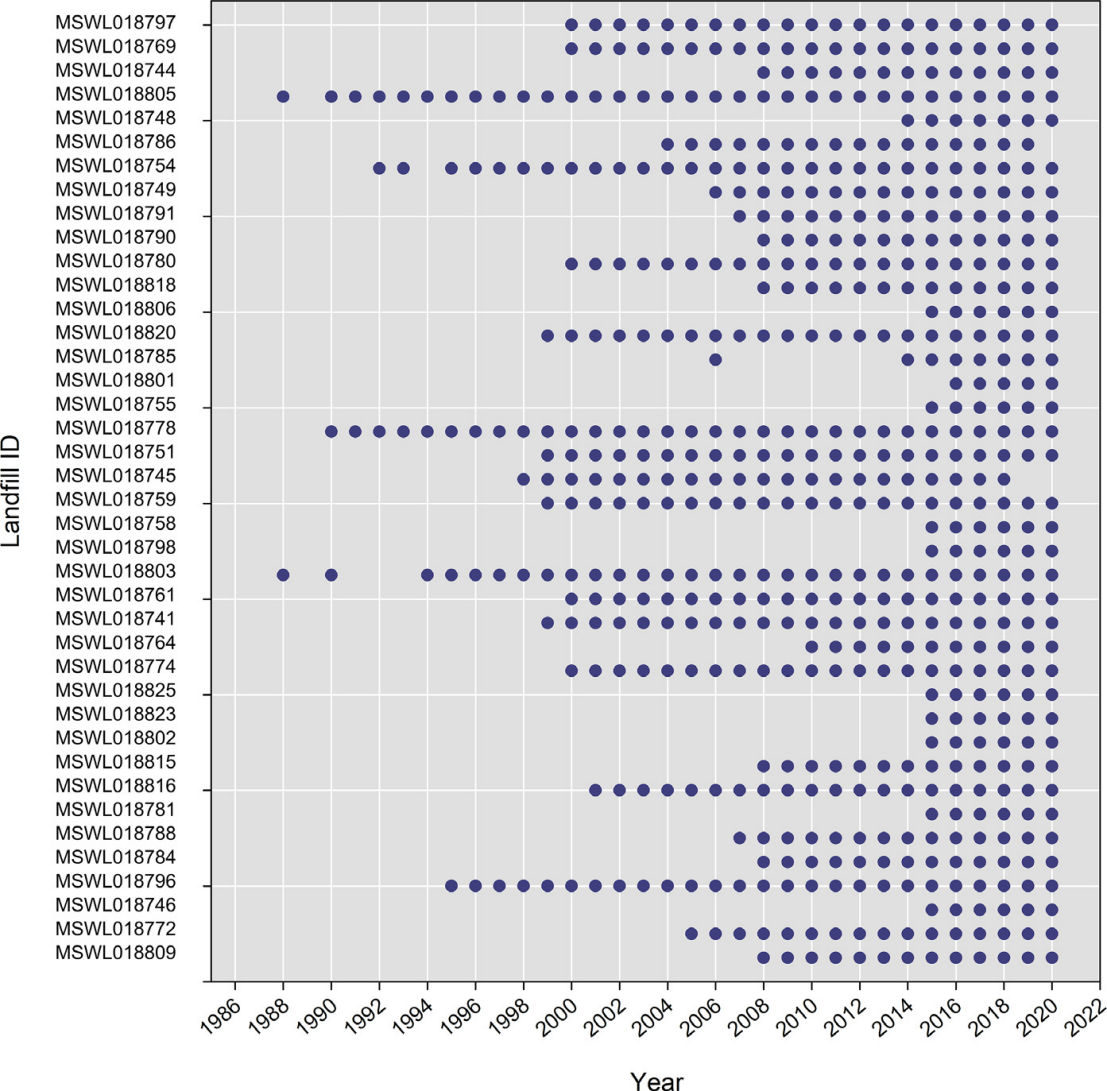
Blue circle symbols indicate the presence of available surface area data in each year for each landfill, identified by its facility identification number.

The surface area data are categorized in a delimited text file (Ohio_Landfill_Surface_Areas.csv), organized under header columns: (A) Year, (B) Landfill Name, (C) Identification Number, (D) Filled Area (Square Feet), (E) Notes, and (F) Source. Fig. 3 presents the distribution of surface area data within Ohio_Landfill_Surface_Areas.csv indicating the presence or absence of data for each landfill by year.

**Fig. 4:**
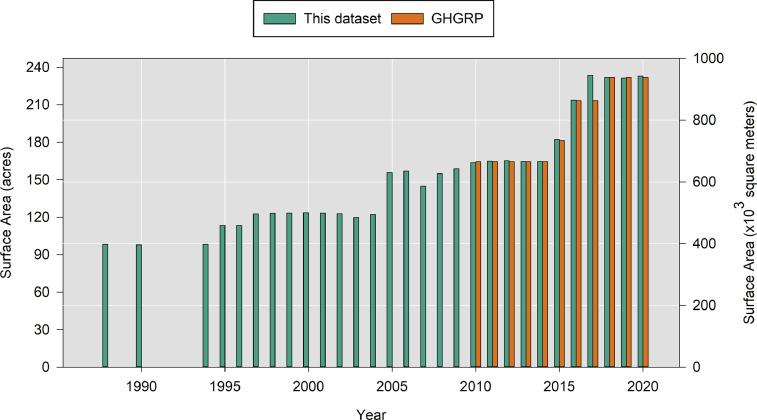
Calculated planar surface areas for Franklin County Landfill compared to the existing GHGRP dataset.

In solid waste engineering, surface area data are used to normalize leachate generation rates amongst larger and smaller landfills, and thus these data have high utility. In 2020, the smallest active landfill was Bond Road Landfill at 20.5 acres (8.31 hectares) and the largest was Carbon Limestone at 404 acres (163 hectares), with an average size of 119 acres (48.3 hectares). As shown in Fig. 4, the information in Ohio_Landfill_Surface_Areas.csv supplements and increases the availability and accessibility of historical information for many of the landfills compared to other existing datasets such as EPA's Greenhouse Gas Reporting Program (GHGRP).

**Fig. 5:**
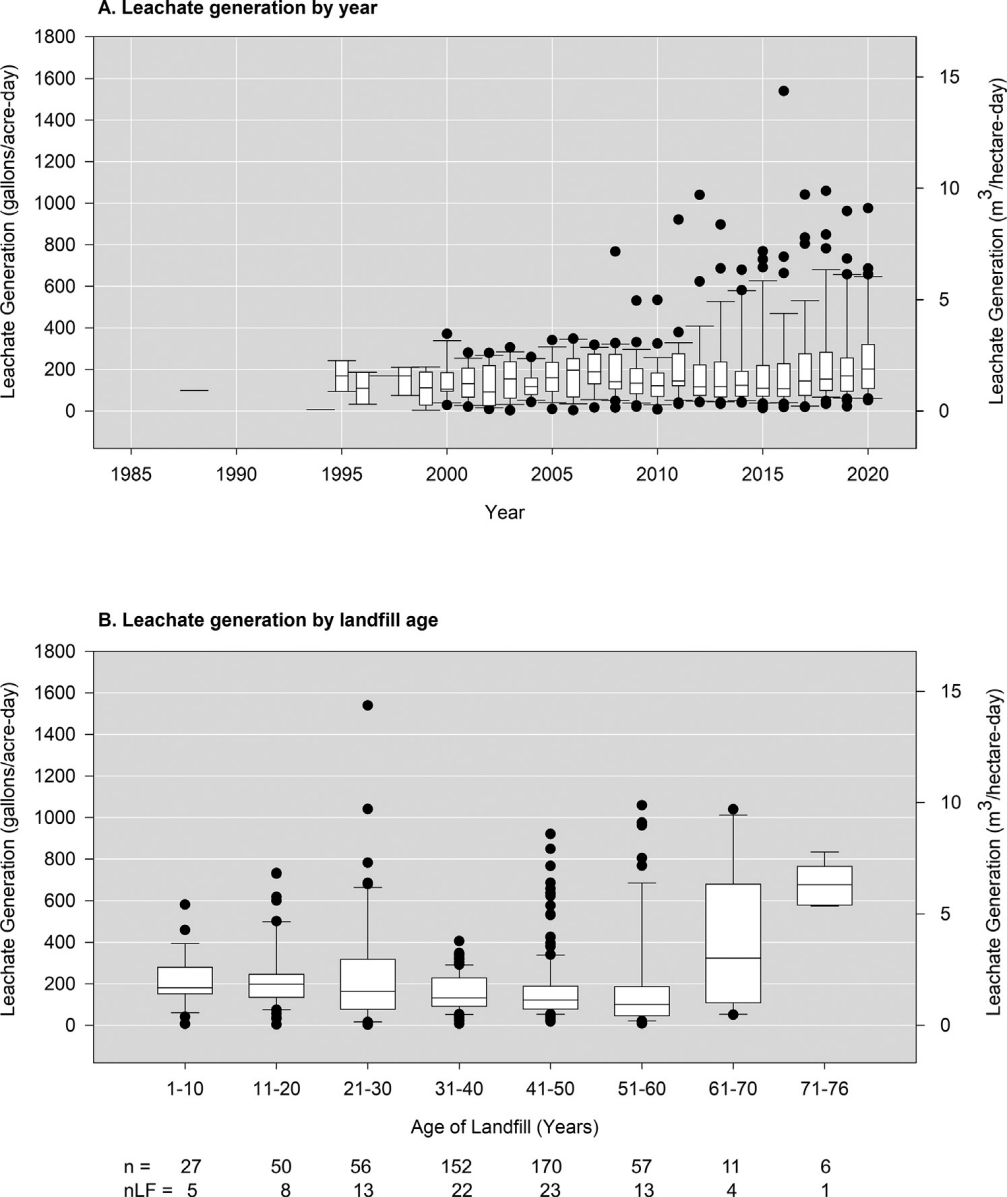
Normalized Ohio landfill leachate generation by (A) year and (B) year of landfill operation. nLF is the number of unique landfills in a year and n is the number of data points in that year.

Fig. 5A shows normalized leachate generation by year using data from Ohio_Leachate_Volumes.csv and Ohio_Landfill_Surface_Areas.csv. Since 2015, average generation increased from 197 to 265 gallons/acre-day in 2020 (1.85 to 2.48 m^3^/hectare-day, respectively). Fig. 5B shows leachate generation with respect to facility age. For closed landfills, leachate generation is expected to decrease over time as water drains and stormwater infiltration is prevented by the final cap. However, for active landfills where new disposal cells are added periodically, leachate generation does not appear to decrease. Although this dataset includes three closed landfills, the oldest landfill here is an active landfill.

**Fig. 6:**
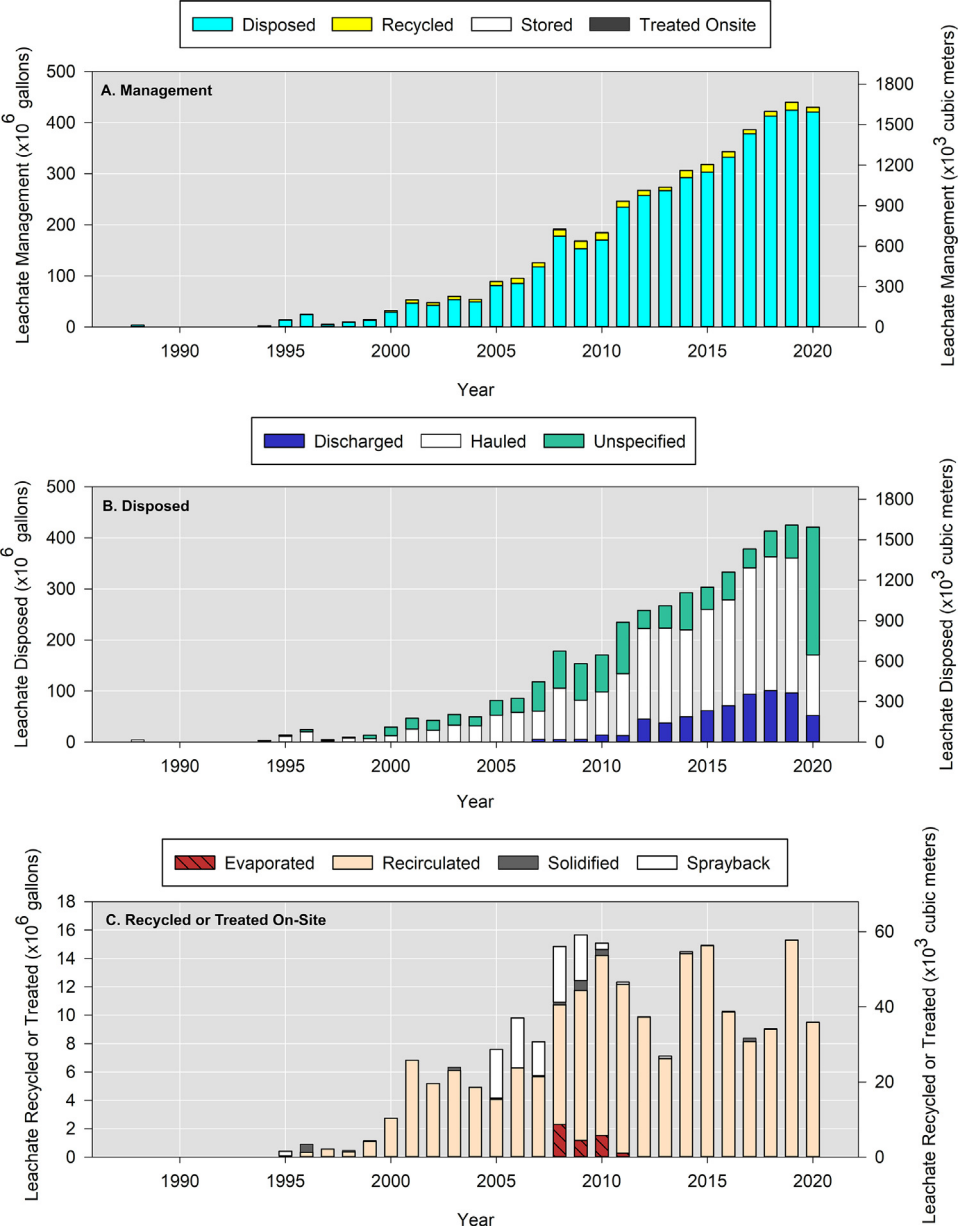
The volumes of leachate (A) managed from 1988-2020. Methods of leachate disposal (B) or on-site recycling and treatment (C).

Using data from Ohio_Leachate_Volumes.csv, Fig. 6 presents leachate management data for 2010-2020 in Ohio including disposal to an offsite facility, on-site treatment, recirculation, and solidification. Note that these data only account for the solidified landfill leachate and do not account for other wastewaters that may be solidified. Solidified leachate was only 0.29-2.94% of the total annual volume recycled and 0.007-0.064% of leachate generated in those years. It is more common to solidify other wastewaters which are a revenue source for landfills that accept them [3]. In total, recycling accounted for only 2.2% of the total managed volume in 2020 representing a 36% decrease from 2015. Fig. 6B illustrates more leachate was disposed by hauling via trucks than discharging via a sewer main. From 2010-2020, 31 landfills reported hauling leachate, 9 landfills reported direct discharge, and 4 landfills reported both methods simultaneously. In 2020, the reporting format changed and the number of landfills that did not specify the disposal method increased significantly. Fig. 6C presents leachate volumes that were recycled between 1992-2020. Recirculation, where leachate is injected into the landfill subsurface, was the most common recycling technique. Sprayback generally refers to a term whereby leachate is sprayed onto the working face (active disposal area).

## 3 Experimental Design, Materials and Methods

### 3.1 Data collection approach

Landfills that accept MSW in the state of Ohio are required to submit annual operational reports to the state's regulatory agency. These annual reports contain useful information regarding leachate (wastewater) generation and where and how this leachate is managed. Annual reports are considered public records and made accessible on Ohio Environmental Protection Agency's (Ohio EPA) public records repository eDocument Management System [4]. From 2019-2021, the eDocument system was queried for annual operational reports from MSW landfills for the years 1988-2020. The individual facilities for which reports were available are presented in Table 2. An attempt was made to collect data from both active and closed MSW landfills. However, closed landfills, which are generally older than active landfills, may not have had leachate collection systems and therefore could not control or collect leachate. Thus, the data are mostly from active MSW landfills. Neither construction and demolition debris landfills nor hazardous waste landfills were included in this effort.

**Table 2:** Landfills included in the leachate database.

Landfill Name	State Landfill ID	Status as of 2020	GHGRP ID	Year Opened	Year Closed	Latitude	Longitude
Adams County	MSWL019993	Closed	N/A	1965	1990	38.77358	-83.5477
Akron Regional	MSWL018777	Closed	1006088	1969	2002	41.1513	-81.5593
American	MSWL018809	Active	1007722	1976	N/A	40.72015	-81.2638
Apex Environmental	MSWL018772	Active	1006777	2005	N/A	40.43333	-80.9112
Athens-Hocking Reclamation Center	MSWL018746	Active	1011217	1983	N/A	39.46652	-82.2777
Beech Hollow	MSWL018796	Active	1004815	1995	N/A	39.13368	-82.4752
Bond Road	MSWL018784	Active	N/A	1965	N/A	39.20658	-84.8172
Brown County	MSWL018788	Active	1002425	1966	N/A	38.89771	-83.9065
Carbon Limestone	MSWL018781	Active	1003102	1963	N/A	40.99863	-80.5285
Celina	MSWL018816	Active	1008004	1971	N/A	40.45618	-84.5633
Cherokee Run	MSWL018815	Active	1002480	1972	N/A	40.40898	-83.7285
City of Brooklyn	MSWL018802	Closed	N/A	1990	2015	41.44548	-81.7512
Coshocton	MSWL018823	Active	1007579	1997	N/A	40.05686	-81.8261
Wyandot County	MSWL018797	Active	1002555	1960	N/A	40.91105	-83.3292
Countywide	MSWL018825	Active	1008003	1991	N/A	40.67835	-81.4252
Crawford County	MSWL018774	Active	1002276	1969	N/A	40.15143	-82.8706
Defiance County	MSWL018764	Active	1001101	1969	N/A	41.24782	-84.4126
Erie County	MSWL018741	Active	1006103	1969	N/A	41.3439	-82.5985
Evergreen	MSWL018761	Active	1007723	1973	N/A	41.60309	-83.5034
Franklin County	MSWL018803	Active	1003787	1985	N/A	39.5568	-84.3043
Gallia County	MSWL018798	Active	1007665	1978	N/A	38.98485	-82.2391
Geneva	MSWL018758	Active	1003392	1980	N/A	41.79298	-80.9096
Glenwillow	MSWL019366	Closed	1006345	1940	1997	41.3568	-81.4638
Hancock County	MSWL018759	Active	1003638	1969	N/A	41.11484	-83.6793
Henry County Commissioners	MSWL018745	Closed	1000397	1968	2013	41.33586	-84.0758
Hoffman Road	MSWL018751	Active	1002581	1975	N/A	41.70154	-83.509
Kimble	MSWL018778	Active	1004399	1960	N/A	40.56773	-81.0232
Lake County	MSWL018755	Active	1005878	1976	N/A	41.79298	-80.9096
Lorain County II	MSWL018801	Active	1007969	1972	N/A	41.30622	-82.1725
Mahoning	MSWL018785	Active	1007756	1969	N/A	40.91845	-80.5819
Muskingum	MSWL019999	Closed	N/A	1985	1993	N/A	N/A
Noble Road	MSWL018820	Active	1004850	1997	N/A	40.96882	-82.4826
Pike Sanitation	MSWL018806	Active	1006383	1987	N/A	39.08238	-82.9561
Pine Grove Regional Facility	MSWL018818	Active	1003105	1981	N/A	39.58096	-82.7195
Port Clinton/Ottawa County	MSWL018780	Active	1004484	1974	N/A	41.52523	-83.0323
Preble County	MSWL018790	Active	1003571	1971	N/A	39.68774	-84.6424
Rumpke	MSWL018791	Active	1004856	1945	N/A	39.27489	-84.5982
Stony Hollow	MSWL018749	Active	1007811	1996	N/A	39.71562	-84.2485
Suburban	MSWL018754	Active	1003661	1982	N/A	39.91019	-82.2484
Sunny Farms	MSWL018786	Active	1007748	1970	N/A	41.09102	-83.4143
Tunnel Hill Reclamation	MSWL018748	Active	1010596	2007	N/A	39.73526	-82.1568
Williams County	MSWL018805	Active	1003695	1969	N/A	41.51793	-84.5865
Wilmington	MSWL018744	Active	N/A	1960	N/A	39.44011	-83.855
Wood County	MSWL018769	Active	N/A	1971	N/A	41.37711	-83.7342

### 3.2 Leachate transcription process

**Fig. 7:**
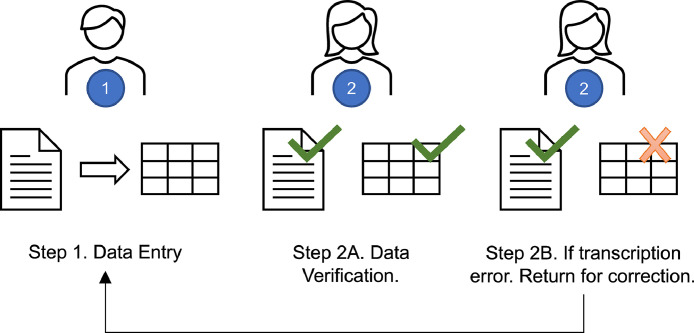
Infographic of data transcription and verification process.

Landfill operators submit annual hardcopy reports to Ohio EPA which include, but are not limited to, monthly volumes of leachate disposed, recycled, or treated on-site. Ohio EPA personnel review and then scan the reports for recordkeeping into the eDocument Management System. Landfill leachate volumes (gallons) were manually transcribed from electronic documents (.pdf) into separate delimited text files (.csv). Transcribed values were independently verified in a round-robin format, as presented in Fig. 7 (i.e., the data is checked for accuracy by a second individual). Data verification was performed by visually comparing the original (.pdf) to the transcribed values (.csv). During the leachate data verification step, 2,304 entries (23% of the 9,985 data points) were verified. Of those, 12 entries were found to contain transcription errors, and these were corrected.

### 3.3 Note on leachate data quality

In practice, leachate is collected into multiple pump stations across the landfill and held in one or more central holding tanks or ponds. Flow meters can utilize different technologies to measure the rate or volume of liquid over time. The types of flow meters are not identified in the reports but are used throughout the industry for this purpose and are understood to have adequate accuracy and precision. The values/data are reported by the landfill operator or their representative and the annual reports are reviewed for accuracy by Ohio EPA personnel. Leachate volumes are required to be included in the reports but do not have regulatory “triggers” and thus, any value can be reported. Variation is expected because of seasonal weather and ongoing landfill construction. Thus, all values reported are assumed to be accurate for research purposes.

### 3.4 Surface Area Measurement Process

Topographic maps which display property boundaries and limits of waste placement are included in the annual reports. Scales embedded within the maps were used to determine the planar (2-dimensional) surface areas with measurement tools in Adobe Acrobat Pro [5]. The perimeter of existing waste placement was determined using the map legends. Points were placed around this perimeter to create an enclosed feature and area was calculated by the software. In cases where the determined area did not comport with previous years’ values (50% larger or smaller), or in cases where topographic maps were not included in the scanned versions of the annual reports, Google Earth Pro's Historical Imagery feature was used [6]. Google Earth Pro's Historical Imagery tool has consistent aerial images available starting in the 1980s. Aerial images of the landfill from that year were located, perimeters of waste placement were determined, and the embedded area measuring tool was used to determine area of waste placement. If a topographic map was not included in the report and a historical image of the site did not exist for that calendar year, then no surface area entry was created. The surface area data were reviewed as represented in Fig. 7, with a second individual repeating the process to reproduce similar results. During the surface area data verification step, 87 entries (14% of the 610 data points) were verified. Of those, 35 were found to be developed in error due to document scaling issues and were corrected.

### 3.5 Note on surface area data quality

Many landfills report surface area data to US EPA's GHGRP as well as Ohio EPA [7]. The validity of this data was checked by comparison to GHGRP data. From 2010-2020, the years for which the datasets overlap, the average percent difference of surface area was 4-9% higher or lower than the GHGRP dataset, indicating an acceptable level of accuracy given the approach taken. This comparison is attached as a Supplemental Material to this manuscript.

## Ethics Statements

This work did not involve human subjects, animal experiments, or any data collected from social media platforms. The data are derived from public records published by the State of Ohio and do not contain personal or confidential information.

## CRediT authorship contribution statement

**Max J. Krause:** Conceptualization, Supervision, Writing – original draft. **Natalie Detwiler:** Data curation, Validation, Writing – original draft. **William Eades:** Data curation, Validation. **Davin Marro:** Data curation, Validation. **Amy Schwarber:** Validation, Writing – review & editing. **Thabet Tolaymat:** Project administration, Writing – review & editing.

## Declaration of Competing Interest

The authors declare that they have no known competing financial interests or personal relationships that could have appeared to influence the work reported in this paper.

## Data Availability

Ohio municipal solid waste landfill leachate volumes and surface areas (Original data) (Mendeley Data).
